# Spinocerebellar ataxia 28: a novel AFG3L2 mutation in a German family with young onset, slow progression and saccadic slowing

**DOI:** 10.1186/s40673-015-0038-7

**Published:** 2015-12-16

**Authors:** Christine Zühlke, Barbara Mikat, Dagmar Timmann, Dagmar Wieczorek, Gabriele Gillessen-Kaesbach, Katrin Bürk

**Affiliations:** Institute of Human Genetics, University of Lübeck, Lübeck, Germany; Institute of Human Genetics, University of Duisburg-Essen, Essen, Germany; Department of Neurology, University of Essen, Essen, Germany; Department of Neurology, Philipps University of Marburg, Baldinger straße, 53043 Marburg, Germany; Paracelsus-Elena Klinik, Kassel, Germany

**Keywords:** AFG3L2, Spinocerebellar ataxia, Slow saccades

## Abstract

**Background:**

Spinocerebellar ataxia type 28 (SCA28) is related to mutations of the *ATPase family gene 3*-*like 2* gene (*AFG3L2*). To date, 13 private missense mutations have been identified in families of French, Italian, and German ancestry, but overall, the disorder seems to be rare in Europe. Here, we report a kindred of German ancestry with four affected family members presenting with slowly progressive ataxia, mild pyramidal tract signs and slow saccades.

**Methods:**

After excluding repeat expansions in the genes for SCA1-3, 6-8, 10, 12, and 17, Sanger sequencing of the coding regions of *TTBK2* (SCA11), *KCNC3* (SCA13), *PRKCG* (SCA14), *FGF14* (SCA27) and AFG3L2 (SCA28) was performed. The 17 coding exons of AFG3L2 with flanking intronic sequences were amplified by PCR and sequenced on both strands.

**Results:**

Sequencing detected a novel potential missense mutation (p.Y689N) in the C-terminal proteolytic domain, the mutational hotspot of AFG3L2. The online programme “PolyPhen-2” classifies this amino acid exchange as *probably damaging* (score 0.990). Similarly to most of the published SCA28 mutations, the novel mutation is located within exon 16. Mutations in exon 16 alter the proteolytic activity of the protease AFG3L2 that is highly expressed in Purkinje cells.

**Conclusions:**

Genetic testing should be considered in dominant ataxia with pyramidal tract signs and saccadic slowing.

## Background

The spinocerebellar ataxias (SCAs) represent a clinically and genetically heterogeneous group of inherited neurological disorders with overlapping as well as highly variable phenotypes characterised by progressive incoordination, dysarthria and impaired eye movements. To date, more than 30 genetic loci have been described [1]. Mutations have been isolated in 20 genes, so far. Ten SCAs are caused by repeat expansions while deletions, missense, nonsense or frame shift mutations have been identified in the remaining genes. The genetic locus for SCA28 had been mapped to chromosome 18 in 2006 [2]. Meanwhile, 13 missense mutations of the *ATPase family gene 3*-*like 2* gene (*AFG3L2*) have been reported to cause ataxia [3–7]. In addition, homozygous *AFG3L2* mutations were identified in a spastic ataxia-neuropathy syndrome [8]. Here, we describe a family of German ancestry carrying a novel AFG3L2 mutation.

## Results and discussion

### Clinical findings

Onset was subtle with first symptoms not always indicative for hereditary cerebellar ataxia: the 81 year old mother first noticed bilateral ptosis at the age of 55 that worsened over time and required blepharoplastic surgery. Patient 3 suffers from epilepsy with infrequent seizures since adolescence.

Progression was usually slow with most patients remaining ambulatory several decades after onset. Saccadic slowing developed over time. For further clinical details, see Table 1. MRI studies yielded isolated cerebellar atrophy with intact brain stem and cortical structures. In two patients, there was evidence for additional small-sized white matter lesions on T2 weighted MRI images that could not be explained by vascular or inflammatory disease. Electrophysiological studies in patient 3 yielded normal motor and sensory nerve conduction velocities and amplitudes (peroneal, tibial and sural nerve).

**Table 1:** Clinical features

	Patient 1	Patient 2	Patient 3	Patient 4
Sex	F	M	F	M
Age	81	61	59	53
Age of onset and First symptoms	55 ptosis + falls 60 gait disturbance	24 gait disturbance	16 seizures 39 gait disorder	no subjective complaints
Walking assistance (age)	65	60	50	−
Disease duration (from onset of gait disorder to physical examination)	36	37	20	?
MRI	Nd	Cerebellar atrophy Discrete white matter lesions (age 56)	Cerebellar atrophy Discrete white matter lesions (age 56)	Nd
Ataxia of stance and gait	+++	++	++	+
Upper limb ataxia	+	+	+	+
Lower limb ataxia	+	+	+	+
Intention tremor	−	−	−	−
Dysarthria	(+)	++	+	+
Impaired smooth pursuit	Cannot be evaluated due to limitation of gaze	+	−	+
Gaze evoked nystagmus horizontal	Cannot be evaluated due to limitation of gaze	+	−	+
Impaired suppression of the vestibuloocular reflex (VOR)	−	−	+	−
Limitation of gaze	Vertical complete Horizontal incomplete	−	−	−
Saccadic slowing	++	−	++	−
Ptosis	+++	−	−	−
VI paresis	−	−	bilateral	−
Dysphagia	−	+	−	−
Hearing loss	+	−	−	−
Deep tendon reflexes	brisk	brisk	increased	brisk
Extensor plantar responses	−	−	−	−
Spasticity	−	−	−	−
Impaired noci- and thermoception	−	−	+	+
Impaired vibration sense	+	+	+	+
Other symptoms	−	sleep apnea	rare seizures	−
SARA score [15]	11/40	11/40	19/40	8/40

### Molecular genetic analysis

Sequencing revealed the heterozygous mutation c.2065T>A in exon 16 of the *AFG3L2* gene in all affected family members. The mutation segregated with the disease. On the amino acid level, this substitution results in the missense exchange p.Y689N.

### Discussion

The clinical presentation in this kindred is highly compatible with the SCA28 phenotype with a slowly progressive cerebellar syndrome, hyperreflexia in the lower limbs (76 %) and saccadic slowing (50 %) [3, 4]. Saccadic slowing has been considered a typical clinical feature in SCA2 while pyramidal tract signs are usually absent [9, 10]. Regarding the small sample size, further studies will be necessary to corroborate the combination of slow saccades and pyramidal tract signs as core features of SCA28. Based on these findings, we have actually suspected SCA28 in an individual with early onset and slowly progressive ataxia, saccadic slowing and pyramidal tract signs. Despite a negative family history, molecular genetic testing has actually revealed a SCA28 mutation described earlier by Di Bella and coworkers [5]. Other less characteristic features include ptosis (48 %) and impaired proprioception (45 %) [3, 6]. Behavioural abnormalities and cognitive impairment have been observed in some patients. SCA28 symptoms usually start in early adulthood (mean 39 years, SD 13) with a wide range from 3 to 60 years. Actually, the age of onset seems to depend on the individual mutation [3, 4, 6].

The *AFG3L2* gene, mutated in SCA28, is composed of 17 exons coding for a protein of 797 amino acids with different functional domains: an AAA consensus sequence together with an ATP/GTP-binding site, a peptidase M41 domain containing the HEXXH motif which is a characteristic feature of a zinc-dependent binding domain, and a RNA-binding region [11]. AFG3L2 is highly homologous to paraplegin, the product of the *SPG7* gene. Mutations in the *SPG7* gene are responsible for a subtype of hereditary spastic paraplegia (HSP) [12]. Both, AFG3L2 as well as paraplegin are metalloproteases of the AAA-superfamily; as components of the two mitochondrial AAA (m-AAA) protease isoenzymes in the inner mitochondrial membrane they are involved in the degradation of non-assembled membrane proteins as well as in the activation of mitochondrial proteins [13]. Notably, 12 of 13 published SCA28 mutations correspond to missense exchanges with 11 mutations being located within exon 16 which contributes to the peptidase M41 domain [4–7]. They alter the proteolytic activity of the protease AFG3L2 that is highly expressed in Purkinje cells [5]. The resulting mitochondrial impairment might account for the clinical similarities of SCSA28 and SPG7 with mitochondrial disorders. Two or even three different mutations affecting amino acids at positions 666, 671, and 689 have been identified in SCA28 patients to date. The variation p.Y689N (c.2065T>A) present in our family, is not listed in the integrated map of genetic variation from 1092 human genomes (1000genomes.org). Interestingly, another mutation affecting the tyrosine residue at position 698 (p.Y689H) has recently been identified in another SCA28 patient [7].

## Conclusion

Based on these findings, the missense mutation p.Y689N is likely to have a pathogenic impact on the SCA28 phenotype. This assumption is supported by the strong conservation of tyrosine (Y) at position 689 e.g. in monkey, mouse, dog, elephant, opossum, chicken and zebrafish (UCSC Genome Browser, hg19). Furthermore, the online programme “PolyPhen-2” classifies this amino acid exchange as *probably damaging* (score 0.990).

## Methods

### Subjects

Clinical data and blood samples were obtained in four affected individuals (mother: patient 1, three of four children: patients 2 to 4, details see Table 1) and one unaffected sibling (57 years at examination, SARA score 0/40, personally examined by DT). The study was approved by the Institutional review board of University of Lübeck.

### Genetic analysis

After having obtained informed consent, genomic DNA was extracted from peripheral blood leukocytes by standard protocols. According to the EFNS guidelines [14] prior to sequence analysis, expansions at the loci for SCA1, 2, 3, 6, 7, 8, 10, 12, and 17 were excluded. Additional Sanger sequencing of the coding regions of *TTBK2* (SCA11), *KCNC3* (SCA13), *PRKCG* (SCA14), and *FGF14* (SCA27) identified only known polymorphisms and SNPs. For AFG3L2, the 17 coding exons with flanking intronic sequences were also amplified by PCR and sequenced on both strands. Pathogenic mutations in SPTBN2 (SCA5), KCND3 (SCA19/22), PDYN (SCA23), and ITPR1 (SCA29) genes have not tested in this kindred but a pathogenic impact appears less likely for one of these genes with respect to phenotype characteristics and geographical restrictions (at least in some of these genotypes).

### Data bases

Ensembl AFG3L2 ENSG00000141385, transcript ENST00000269143. HGMD biobase: www.hgmd.cf.ac.uk; UCSC genome browser: http://genome.ucsc.edu; 1000 genomes: www.1000genomes.org; PolyPhen-2–prediction of functional effects of human nsSNPs: http://genetics.bwh.harvard.edu/pph2/.
